# Digital assessment of falls risk, frailty, and mobility impairment using wearable sensors

**DOI:** 10.1038/s41746-019-0204-z

**Published:** 2019-12-11

**Authors:** Barry R. Greene, Killian McManus, Stephen J. Redmond, Brian Caulfield, Charlene C. Quinn

**Affiliations:** 1 Kinesis Health Technologies Ltd, Dublin, Ireland; 2 Insight Centre for Data Analytics, University College Dublin, Dublin, Ireland; 3 School of Electrical and Electronic Engineering, University College Dublin, Dublin, Ireland; 4 Department of Epidemiology and Public Health, University of Maryland School of Medicine, Baltimore, MD USA

**Keywords:** Public health, Risk factors, Geriatrics, Biomedical engineering

## Abstract

Falls are among the most frequent and costly population health issues, costing $50bn each year in the US. In current clinical practice, falls (and associated fall risk) are often self-reported after the “first fall”, delaying primary prevention of falls and development of targeted fall prevention interventions. Current methods for assessing falls risk can be subjective, inaccurate, have low inter-rater reliability, and do not address factors contributing to falls (poor balance, gait speed, transfers, turning). 8521 participants (72.7 ± 12.0 years, 5392 female) from six countries were assessed using a digital falls risk assessment protocol. Data consisted of wearable sensor data captured during the Timed Up and Go (TUG) test along with self-reported questionnaire data on falls risk factors, applied to previously trained and validated classifier models. We found that 25.8% of patients reported a fall in the previous 12 months, of the 74.6% of participants that had not reported a fall, 21.5% were found to have a high predicted risk of falls. Overall 26.2% of patients were predicted to be at high risk of falls. 29.8% of participants were found to have slow walking speed, while 19.8% had high gait variability and 17.5% had problems with transfers. We report an observational study of results obtained from a novel digital fall risk assessment protocol. This protocol is intended to support the early identification of older adults at risk of falls and inform the creation of appropriate personalized interventions to prevent falls. A population-based approach to management of falls using objective measures of falls risk and mobility impairment, may help reduce unnecessary outpatient and emergency department utilization by improving risk prediction and stratification, driving more patients towards clinical and community-based falls prevention activities.

## Introduction

Population health approaches to the management of major societal healthcare challenges have received much attention of late, with the rise of big data and advances in health assessment technology. Population level preventative screening has the potential to significantly reduce costs and improve long-term outcomes for a variety of conditions. Globally, falls are the most frequent cause of accidental death and disability, thought to cost $50bn each year in the United States^1^ and €25bn each year in the European Union.^2^ Falls can be underreported, with the first presentation of a patient to clinical services only after the first fall has occurred.^3^ Extensive research has shown that falls are not inevitable and can be reduced by 20–50% by appropriate early intervention.^4,5^ However, effective early intervention is contingent on accurate screening and identification of those people at risk of experiencing a fall, ensuring timely and appropriate referral of those patients at higher risk.

Assessment of functional mobility is a critical element in quantifying and understanding the nature of falls risk, and in measuring response to remedial interventions in the assessment and management of falls risk and frailty.^6^ A variety of functional mobility assessments are available and commonly used in research; they include the Timed Up and Go (TUG) test,^7^ which has been shown to be a valid and reliable functional test.^8^ Persons taking longer to complete the TUG test have been shown to have high rates of falls, frailty, and other clinical conditions.^9^ However, research has shown the TUG test has only limited accuracy in predicting falls^10,11^ in older adults. Similarly, slow walking speed has been found to have strong negative associations with a number of long-term outcomes such as dementia, frailty, and longevity.^12^

Objective assessment of falls risk, frailty, and mobility, using a reliable functional test combined with wearable sensors and predictive algorithms supports the assessment by lower cost and lower skilled personnel or clinical personnel without specialist expertise, and may help improve the precision of clinical assessment and referral; this can increase the efficiency of primary care by using standardized assessments that can be administered by a healthcare assistant, as well as reducing inappropriate referrals. Moreover, deployment of community screening technologies may reduce unnecessary outpatient visits and health care utilization by driving medium and low risk patients away from hospitals towards community falls prevention activities. Such an approach could reduce the instances of escalation and emergency acute admissions through increased prevention arising from improved screening, triage, and onward referral of at-risk patients.

A number of recent studies have examining depth camera^13^ and sensor-based^14–20^ systems for assessing gait, mobility and fall risk in older adults. Sensor-based methods for assessing fall risk are often use prescribed motor tasks (e.g. TUG or Five Time Sit to Stand), instrumented with inertial sensors. In a majority of studies, each patient’s self-reported history of falling (cross-sectional fall data) prior to assessment^16^^,19^ is used to validate the reported algorithm, while for a subset of studies each patient was followed-up and their falls tracked for a period of time after the assessment is performed; these fall data were then used to validate the methods’ performance in predicting falls (prospective fall data)^14^^,20^^,21^. Previous research from our group^22^ has reported that fall history data were more accurate in classifying healthy controls than 2 years prospective data (incidents of falls during a follow-up period). Recent studies^23^^,24^ have pointed out flaws in the methodologies of many reported studies which aim to assess fall risk and/or predict falls. Specifically, they noted insufficient validation and inappropriate use of statistical modeling methods in many of the studies reported to date.

The goal of this research is to report results for the deployment in routine clinical practice of a novel digital assessment protocol and algorithm for prediction of falls, frailty and mobility impairment; developed, trained and validated by our team over the past 11 years. We report an observational study of the performance of these algorithms, tested on a large statistically independent sample, in diverse real-world clinical settings over a period of 5 years.

## Results

**Fig. 1:**
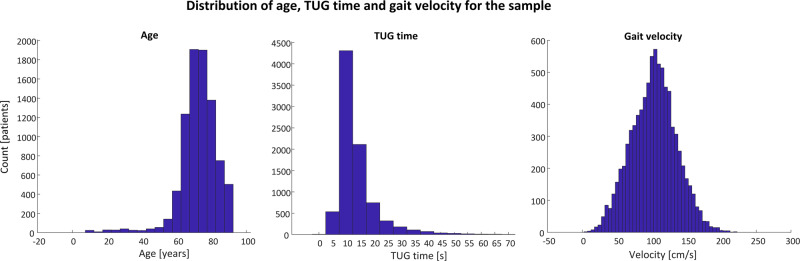
Histograms of age (first panel), TUG time (second panel) and gait velocity (third panel) for all participants included in the sample.

A total of 14,611 records from 8521 participants (5415 female, 3106 males) were available for analysis. Where multiple records were available per participant, only the first record was included in analysis. The mean age of the sample was 72.7 ± 10.7 years, with mean height and weight of 165.9 ± 10.4 cm and 73.8 ± 15.9 kg, respectively. The mean TUG time was 13.9 ± 7.4 s, while the mean gait velocity (during the TUG test) was 101.9 ± 32.5 cm/s (see Table 1). Figure 1 shows the distributions of the age, TUG time and gait velocity for the cohort.

Data were obtained from 38 healthcare organizations from six countries (UK, USA, Ireland, Canada, Australia and Hungary). The breakdown of the sample of participants per country is as follows: UK—29.5%, Ireland—41.8%, USA—11.1%, Canada—15.2%, Others—2.2%. Organizations were divided into five categories as follows: Academic (3.2%), Primary care (91.4%), Residential care (1.3%), Secondary care (<1%), Senior living (3.2%). Users conducting each patient assessment for each organization were grouped into four categories as follows: care assistant (40.3%), Physician (<1%), physical therapist (55.9%) and researcher (3.0%). Similarly, the patient profiles assessed by each organization were grouped into six categories as follows: community dwelling older adults (88.3%), neurological patients (1.4%), rehabilitation patients (7.1%), residential care (2.0%), and unknown patient type (1.2%).

### Questionnaire data

A total of 11,635 questionnaire records from 6950 participants were available for analysis, as above only the first record with a completed questionnaire for each participant was included in analysis. 1779 participants (25.6%) reported a history of falls in the previous 12 months with 4477 falls reported in total (average of 0.65 falls per participant and 2.51 falls per faller). 855 participants reported recurrent falls. Table 2 details the prevalence of each of the reported clinical risk factors per patient group and organization type.

**Table 1:** Demographic information for the sample (*N* = 8,521), broken down by patient type.

	Community dwelling	Neurological	Rehabilitation	Residential care	Unknown
N (F/M)	4766/2755	58/64	410/196	119/49	58/41
Height (cm)	165.9 ± 9.9	168.1 ± 7.0	166.1 ± 10.5	163.1 ± 9.9	162.7 ± 22.7
Weight (kg)	73.5 ± 15.1	73.9 ± 9.6	80.1 ± 21.3	68.8 ± 12.2	67.8 ± 27.0
Age (years)	73.9 ± 8.1	62.6 ± 12.3	62.117.8	80.5 ± 8.5	42.8 ± 27.4
TUG time (s)	14.0 ± 7.2	9.6 ± 4.0	12.8 ± 8.0	18.8 ± 11.7	9.0 ± 3.8
Gait velocity (cm/s)	100.6 ± 31.1	131.9 ± 28.7	125.2 ± 36.2	87.8 ± 32.2	132.3 ± 32.9

Data are expressed as mean ± std. dev

Polypharmacy was present for 3240 participants (46.6%), while mobility problems were reported by 2346 participants (33.7%). Vision problems were reported in 2262 records (32.5%), while dizziness while standing up (orthostatic hypotension) was reported by 547 participants (7.9%). A change in ability to manage routine activities in the home (a marker of a person’s ability to live independently) was reported by 1352 participants (19.4%).

### Falls Risk Estimate

For the sensor based FRE (FRE_sensor_), 554 of 8521 participants were excluded due to missing data, leaving 7,967 valid participant records for analysis. Missing data arose as the FRE is not calculated for participants under 60 years of age or if “Falls mode” on the software is turned off. Similarly, for FRE_combined_ and FRE_clinical_, 6,543 participants were included in the analysis, missing data arose for participants under 60 (no value calculated) and when the user chose to skip the questionnaire. The lower limit for age in the calculation of the FRE and FE is enforced due to the fact that the classifier models included in the software were trained and validated on a cohort of persons 60 years of age and older. Table 4 details the prevalence of high falls risk per organization type and patient type.

**Fig. 2:**
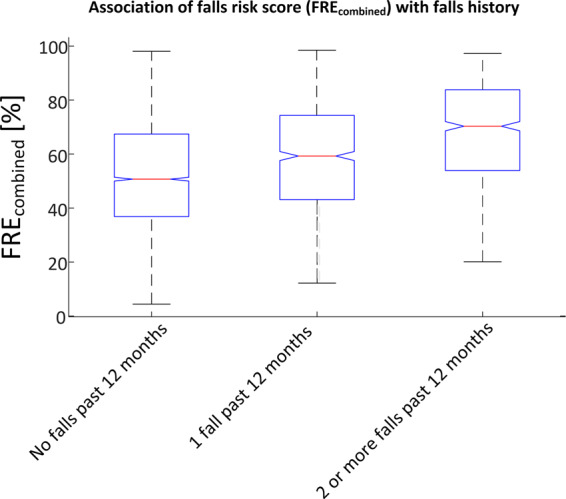
Association of falls risk score (FRE_combined_) with falls history (*F* = 214.19, *p* < 0.0001). The falls risk scores were grouped by no falls in the past 12 months, one fall in the past 12 months and two or more falls in the past 12 months. For each box, the central mark indicates the median, and the bottom and top edges of the box indicate the 25th and 75th percentiles, respectively. The whiskers extend to the most extreme data points not considered outliers, while outliers are denoted individually by ‘ + ’.

6295 participants had both valid FRE data and completed questionnaire data for the first record. 4693 of these participants reported no falls in the previous 12 months, 1008 (21.5%) of these were found to be at high risk or very high risk of falls (based on FRE_combined_). Conversely, 745 of these participants reported two or more falls in the previous 12 months, 153 (20.5%) of these participants were found to be at low risk of falls. FRE_combined_ was found to be significantly associated with falls history (*F* = 214.19, *p* < 0.0001), see Fig.2.

### Frailty estimate

Valid records for 7536 participants were available for FE_sensor_, missing data arose as this measure was introduced in a later software version (2015) and so were not calculated for all participants over the study period. Similarly, no FE values were calculated for participants under 60 or if “Falls mode” was turned off. FE_clinical_ and FE_combined_ were introduced in 2017, this fact along with the ability of the user to skip the questionnaire meant there were valid records for only 3016 participants for these measures. Table 3 details the prevalence of participants in each risk category for each of the FRE and FE scores by participant and organization type.

**Table 2:** Prevalence (%) of clinical fall risk factor per organization type and patient type (*N* = 6950).

	N (valid)	Falls history	Polypharmacy	Vision problems	Dizziness	Mobility problems	Foot problems	Problems with routine activities
All data	6950	25.6	46.6	32.5	7.8	33.7	28.8	19.4
Organization type								
Primary care	6592	25.4	46.4	32.7	8.0	33.7	29.0	19.7
Academic	133	21.8	46.6	27.1	6.0	19.5	14.3	13.5
Senior living	118	39.8	55.1	28.8	4.2	50.0	43.2	19.5
Residential care	91	20.9	52.7	34.1	6.6	30.8	16.5	8.8
Secondary care	16	43.8	50.0	37.5	6.3	50.0	12.5	37.5
Patient type								
Community dwelling	6169	25.2	45.6	33.2	8.4	31.5	27.1	17.1
Rehabilitation	557	31.2	57.1	25.7	2.3	62.1	53.1	48.5
Residential care	141	25.5	62.4	31.9	9.9	28.4	16.3	12.8
Neurological	55	14.5	29.1	32.7	0.0	23.6	18.2	12.7
Unknown	28	15.4	23.1	15.4	0.0	3.8	3.8	3.8

2946 participants had both valid FE data and completed questionnaire data for their first record. 2143 of these participants reported no falls in the previous 12 months. 709 (33.1%) of those who reported no falls were found to be at high risk or very high risk of falls (based on FRE_combined_). Conversely, 387 participants reported two or more falls in the previous 12 months, 79 (20.4%) of these participants were found to be at low risk of falls. FE_combined_ was found to be significantly associated with falls history (*F* = 51.08, *p* < 0.0001).

### Mobility impairment scores

The mobility impairment scores were introduced in 2017 and so values were not calculated for all participants over the study period. From the original 14,611 records for 8521 participants, there were valid mobility impairment scores for 5195 participants. Table 4 details the prevalence of mobility impairment per organization and patient type for each mobility impairment score. Each mobility score was significantly associated with falls history (speed: *F* = 76.78, *p* < 0.0001, turn: *F* = 41.47, *p* < 0.0001, transfers: *F* = 115.46, *p* < 0.0001, symmetry: *F* = 4.13, *p* < 0.05, variability: *F* = 6.23, *p* < 0.01. Four of five mobility scores were significantly associated with self-reported mobility problems (speed: *F* = 261.49, *p* < 0.0001, turn: *F* = 289.04, *p* < 0.0001, transfers: *F* = 547.07, *p* < 0.0001, symmetry: *F* = 3.24, *p* = 0.07, variability: *F* = 9.17, *p* < 0.01).

**Table 3:** Proportion of participants in each risk category for each of the Falls Risk Estimate (FRE) and Frailty Estimate (FE) scores.

Measure	N (valid)	Low risk	Medium risk	High risk	Very high risk
FRE_combined_	6543	2857 (43.7%)	1957 (29.9%)	1467 (22.4%)	262 (4.0%)
FRE_sensor_	7967	3940 (49.5%)	1237 (15.5%)	1080 (13.6%)	1710 (21.5%)
FRE_clinical_	6543	2963 (45.3%)	2112 (32.3%)	1392 (21.3%)	76 (1.2%)
FE_combined_	3016	1075 (35.6%)	822 (27.3%)	915 (30.3%)	204 (6.8%)
FE_sensor_	7536	2553 (33.9%)	1532 (20.3%)	1527 (20.3%)	1924 (25.5%)
FE_clinical_	3016	1324 (43.9%)	962 (31.9%)	673 (22.3%)	57 (1.9%)

## Discussion

To the best of our knowledge this is the largest study reported to date (*N* = 8,521) on either objective measures of falls risk and frailty, or wearable sensor measures of gait and mobility. We report an observational study of a novel digital fall risk assessment protocol that supports the early identification of older adults at risk of falls and provides indications to inform the creation of appropriate personalized interventions to prevent falls. The classifier models used in this protocol were trained prior to deployment on an independent data set. The methods used have been previously validated and shown to be valid,^25,26^ reliable^27,28^ and more accurate than standard methods, in assessing falls^21^^,29–31^ and frailty.^32,33^

A large proportion of patients (range of 22.5–45.8% across measures) were predicted to be at high risk or very high risk for a measure of falls and frailty. These results agree with the epidemiological finding of 1 in 3 older adults being at risk of falling per year.^34^ As expected, a higher prevalence (50.4%) of high or very high falls risk was predicted in residential care settings when compared to primary care and senior living. Similarly, a higher prevalence (46.4%) of high or very high falls risk was predicted in residential care patients when compared to community dwelling older adults.^35,36^ 25.6% of participants reported a fall in the previous 12 months, while 26.4% of participants were predicted to be at high or very high risk of falls (based on FRE_combined_), with 37.1% of participants being at high or very risk of frailty (FE_combined_); this is lower than some international estimates suggest and may reflect the fact that the sample was: (a) healthier than the general population or; (b) fall data were unreliable due to difficulties with underreporting, self-reported recall^3^ or cognitive impairments.^37^ 21.5% and 33.1% of participants who did not report any falls in the previous 12 months were predicted to be at high risk of falls and frailty respectively, which could suggest these are participants with undetected risk who could stand to benefit most from preventative screening programs. 20.5% and 20.4% of participants who reported two or more falls in the previous 12 months were found to be at low risk of falls and frailty, respectively. These participants might be more complex cases, representing a limitation on the accuracy of the assessment tool, where the source of falls risk is not adequately identified by the digital fall risk assessment protocol.

Polypharmacy is considered one of the strongest clinical risk factors for falls^38^ and was reported by 46.6% of participants overall, which is almost twice the rate of self-reported falls, with higher rates among those in residential care (52.7%) and senior living (55.1%). Mobility problems were reported by 33% of participants, while low speed (high risk speed score) was observed in 30% of participants. Higher rates (>50%) of those in senior living and secondary care reported mobility problems as an issue, with a larger proportion of high-risk speed scores observed in residential and secondary care.

We report an average TUG time of 13.9 ± 7.4 s, and an average gait velocity of 102.9 ± 32.5 cm/s. Both measures are in agreement with average values reported in the literature for this population.^10,11,39^ It is worth noting that the proportion of participants with impaired or high-risk symmetry is 2.7%, while 29.8% of patient records indicated slow speed (as calculated against a statistically independent reference data set). This might suggest that asymmetric gait is less prevalent than slow walking speed or high gait variability (19.5%), which is associated with falls.^14^ Problems with transfers (e.g. getting out of a chair), which is associated with poor lower limb and core strength^40^ were found in 17.5% of participants, problems turning (associated with poor balance or vestibular problems^41^) were found in 13.9%. The prevalence of slow walking speed (as determined by the relevant age and gender referenced mobility score) in residential care patients was 35.1% as compared to 18.1% in primary care and 4.8% in senior living. Understanding factors associated with fall risk, such as gait variability or slow walking speed may improve fall risk prevention interventions targeted to specific problems leading to future falls, mobility impairment or frailty.^42^

The reported data have limitations which we have attempted to address; data are from diverse real-world routine clinical settings and so were not controlled research-grade data, data were collected from a cross-section of the population, including population samples which might not be included in a typical research project. As such, some data were missing or were noisy, however, we do not believe there was a systematic or consistent reason for missing data that could affect the results. All users were provided with instruction on test protocol, but we have no way to evaluate treatment fidelity in the routine clinical practice for 38 organizations. We attempted to address this by performing outlier and artifact rejection on the data. In addition, all questionnaire data were self-reported (albeit mediated by the user supervising the delivery of the test and questionnaire). Due to the nature of the population, self-reported data on falls, particularly using retrospective falls history, can be under-reported. While 12 month falls history is a strong predictor of future falls,^43^ consensus guidelines^44^ suggest it is not an appropriate outcome measure for studies of falls and for this reason we have only reported associations with falls history rather than using it to report the predictive accuracy of the reported measures in this independent sample.

This study aims to show the behavior of these previously validated objective measures of falls risk, frailty and mobility impairment obtained from wearable sensor data, on a statistically independent data set from routine clinical settings. We also confirm their expected association with self-reported falls history and self-reported mobility problems.

These data are of crucial importance to clinicians interested in fall prevention as they provide a reliable basis for determining a patient’s falls risk and establishing preventative measures. This protocol which has been shown elsewhere to be cost-effective^45^ for use in fall prevention, broadens the base of users that can perform fall risk assessments. This is because it enables lower skilled or lower-cost staff, such as home care and community workers to perform reliable fall risk assessments in non-clinical environments and allows clinicians such as primary care physicians and physical therapists to interpret these data and improve the precision of referral and diagnosis. In addition, they may allow healthcare managers to estimate demand for services based on target population and expected prevalence of each risk category. This has the potential to improve patient outcomes and significantly reduce the financial burden imposed by falls on global healthcare systems.

## Methods

A sample of 8521 participants were assessed using a digital fall risk assessment protocol (see Table 1). Data included measures of falls, frailty and mobility and were collected between 2014 and 2019. Data were anonymized and stored centrally on a cloud-based server. Each data record contained wearable sensor data and questionnaire responses on clinical fall risk factors.

Each participant provided informed consent, a supervising user consented the participant and collected the data using Kinesis QTUG™ (Kinesis Health Technologies, Dublin, Ireland), a Class I medical device, registered with US, Canadian, EU and Australian competent authorities. Each supervising user was provided with a user guide, an instructional video and additional training if required. Data were acquired in different contexts and pooled for the purposes of the analyses presented in this paper. Some data were obtained as part of research studies which had been approved by the relevant ethics committees and shared with the authors with the consent of study participants. Other data were collected as part of normal clinical practice from a variety of healthcare organizations (including primary care, senior living, secondary hospital care, and residential care) and shared with the authors in adherence with local data governance procedures. Data were anonymized and stored in line with data privacy regulations in each country (e.g. HIPAA, GDPR). Data were obtained from a variety of healthcare organizations including primary care, senior living, secondary (hospital) care, academic research and residential care. Users included individuals from a variety of professional groups, including physical therapists, care assistants and researchers. Each participant carried out the TUG test wearing body-worn inertial measurement units (IMUs) on each leg below the knee. Each IMU was held in place with dedicated reusable Velcro straps or with disposable bandages. Users were asked to instruct participants to complete the TUG test “as fast as safely possible”; participants were asked to stand-up, walk 3 m, turn 180°, walk back to the chair and sit down. Each test takes approximately 5 min, including application of the sensors and explaining the protocol. Data were extracted from the database using MySQL workbench 8.0 (Oracle, CA) and analyzed using Matlab v9.3.0 (Mathworks, Natick, VA).

### Questionnaire data

The supervising user was asked to complete a questionnaire with the participant, asking them questions on their clinical fall risk factors (this questionnaire was optional and could be skipped in the software (for users for whom clinical falls data were not of interest)). The questionnaire (see Supplementary methods) is based on the American and British Geriatric societies (AGS/BGS) guidelines and aims to capture self-reported data on the main clinical risk factors linked to falls in older adults.^46^ The clinical risk factors recorded by the questionnaire include history of falls, polypharmacy, mobility problems, orthostatic hypotension, foot problems, vision problems (vision impairment) and ability to live independently. Participants were asked about their history of falls in the previous 12 months. Falls history was considered in three categories: no falls in the previous 12 months, one fall in the previous 12 month, two or more falls (recurrent faller) in the previous 12 months.

### IMU sensor data

The recorded IMU sensor data are automatically processed immediately upon completion of each TUG test, producing 71 different measures of gait and mobility (QTUG mobility parameters), which are applied to pre-trained classifier models to produce validated statistical predictions of falls risk and frailty.^29–32^ For the purposes of this study we report a subset of these parameters: TUG time, gait velocity, Falls Risk Estimate (FRE), Frailty Estimate (FE), and mobility impairment scores. All data reported here were independent of the data previously used to develop and validate each of the statistical classifier models used (see Supplementary material for further details).

TUG time, the time to complete the TUG test, is a standard measure used to assess fall risk. It is measured from the moment the user says ‘go’ to the moment the participant’s back touches the chair. Traditionally, this is measured using a stopwatch but is captured here using a start/stop button on a tablet (QTUG software). The gait velocity during the TUG test is calculated using a previously reported algorithm.^21^^,47^ For reference purposes, we use a standard cut-off time for the TUG test; with a time greater than 13.5 s^11^ considered high risk.

### Falls risk estimate

Statistical fall risk predictions are calculated using a combination of sensor data, anthropomorphic and questionnaire data applied to classifier models previously trained on independent data.^29^ If a full dataset is available for a participant, three falls risk estimate scores are produced: Sensor-based (FRE_sensor_), Questionnaire-based (FRE_clinical_) and Combined (FRE_combined_).

FRE_sensor_ is based on IMU sensor data and anthropomorphic data only, applied to a regularized discriminant model. FRE_clinical_ is based on questionnaire and anthropomorphic data applied to a logistic regression model. FRE_combined_ is a weighted average of the other two FRE scores. (see Supplementary methods for more details). Prospective and cross-sectional validation data for each model have been reported previously^29,30^ and found that FRE_combined_ is more accurate than both FRE_clinical_ and FRE_sensor_ individually, while FRE_combined_ and FRE_sensor_ have also been shown to be significantly more accurate (approximately 20% depending on the study population) than the TUG time or Berg balance scale (BBS) in assessing falls risk.^21^^,30^ In this observational study, a one-way ANOVA was used to test the association between FRE_combined_ and falls history.

### Frailty estimate

As with the FRE, three frailty estimate (FE) scores—derived using previously validated predictive models for estimating frailty based on the Fried frailty phenotype^48^ (a well-validated standard measure of clinical frailty)—are calculated for each QTUG assessment: Sensor-based (FE_sensor_), Questionnaire-based (FE_clinical_), Combined (FE_combined_).

FE_sensor_ is based on a previously reported algorithm^32^ and uses IMU data and anthropomorphic data applied to a logistic regression model (see Supplementary material for more details). FE_sensor_ was found to be slightly more accurate (4%) than TUG time in assessing frailty but would have the advantage of being faster to administer and suitable for a non-specialist user. FE_clinical_ and FE_combined_ use a similar methodology to FRE_clinical_ and FRE_combined_^29^ which are based on the questionnaire data applied to a logistic regression model and a weighted average of the other two FE scores, respectively (see Supplementary methods for more details). Both are reported here for the first time. In this paper, a one-way ANOVA was used to test the association between FE_combined_ and falls history.

### Mobility impairment scores

Mobility impairment is identified by grouping the calculated QTUG mobility parameters into five functional categories: Speed, Variability, Symmetry, Transfers, and Turning.

Mobility issues per functional category are identified by calculating a percentile score (compared to an independent reference sample^22^^,^^30,31^) for each QTUG parameter per group, with the mobility impairment score taken as the mean of the parameter percentiles within each functional category. Scores are also stratified by age range and gender. Each mobility impairment score can be interpreted as an aggregated measure (between 0 and 100%) of the severity of a person’s impairment in the given functional mobility category, referenced against the population stratified by age range and gender. See Supplementary methods for more details.

**Table 4:** Proportion of participants with high (or very high) falls risk (FRE_combined_, *N* = 6543), frailty (FE_combined_, *N* = 3016) and mobility impairment (N = 5195) per QTUG score by organization and patient type.

	FRE_combined_	FE_combined_	TUG	Speed	Turn	Transfers	Symmetry	Variability
Organisation type								
Residential care	50.44	44.25	58.40	30.1	13.3	20.4	0.0	15.0
Primary care	20.53	13.23	35.00	18.8	8.2	11.1	1.7	12.3
Senior living	15.02	3.30	46.50	4.8	2.9	1.8	0.7	0.7
Academic	9.06	8.70	19.60	11.2	4.7	5.1	0.4	10.5
Secondary care	8.06	6.45	43.50	11.3	8.1	3.2	0.0	8.1
Patient type								
Residential care	46.43	41.67	58.30	35.1	14.9	17.9	0.0	16.1
Community dwelling	20.73	12.41	36.10	18.7	7.5	10.9	1.7	12.0
Rehabilitation	14.36	18.32	27.60	12.7	14.2	8.4	1.0	10.1
Unknown	2.02	4.04	9.10	2.0	1.0	0.0	0.0	10.1
Neurological	1.64	0.00	10.70	3.3	1.6	5.7	0.8	7.4

For reference purposes, the proportion of patients (%) deemed high risk by TUG time (TUG time > 13.5 s) are also included

### Risk categories

The falls risk and frailty scores are essentially posterior probabilities and defined as low risk if less than 50%, 50–69% is considered medium risk, while high risk and very high risk are 70–89% and greater than 90% respectively. These probability definitions are based on empirically derived thresholds obtained from testing on algorithm development datasets.

A mobility impairment score of less than 50% is below the median (50th percentile) and considered low risk, while a score of 50–69% is deemed medium risk. If the mobility impairment score for a given category is above 70% (70th percentile) the participant is considered to display functional impairment in that functional category.

One-way ANOVA was also used to examine the association of mobility scores with falls history as well as with self-reported mobility problems.

### Reporting summary

Further information on research design is available in the Nature Research Reporting Summary linked to this article.

## Supplementary information

Reporting summary checklist

Supplementary material

## Data Availability

The data that support the findings of this study are available from Kinesis Health Technologies Ltd. Restrictions apply to the availability of these data, which were used under license for the current study, and so are not publicly available. Data are however available from the authors upon reasonable request and with permission of Kinesis Health Technologies Ltd.

## Code availability

The methods used to develop the algorithms used in this study have been reported elsewhere^25,29,30,49^ and provide detail required for implementation. The code used to calculate the reported results are available from Kinesis Health Technologies Ltd but restrictions apply to the availability of this code, which is used under license for the current study, and so are not publicly available. Code is however available from the authors upon reasonable request and with permission of Kinesis Health Technologies Ltd.
