# Supplementary material for: Digital assessment of falls risk, frailty, and mobility impairment using wearable sensors
Source: NPJ Digit Med. 2019 Dec 11;2:125. doi: 10.1038/s41746-019-0204-z (PMC6906412; doi:10.1038/s41746-019-0204-z)
Supplement: Supplementary file 2 — Supplementary material [file 41746_2019_204_MOESM2_ESM.pdf]

Supplementary material

Supplementary Methods

Digital fall risk assessment protocol

The protocol consists of a simple mobility assessment, the Timed Up and Go (TUG) test and a questionnaire on self-reported questionnaire data on clinical fall risk factors.

The TUG test is a standard functional test of mobility, commonly used in clinical practice to screen for gait and balance issues in the older adult population[1-3]. The test requires the participant to rise from a chair, walk three metres, turn through 180º at a designated spot, return to the seat and sit back down. The time taken to perform the test is recorded by a supervising user.

Falls risk questionnaire

The AGS/BGS offer guidelines aimed to capture the main clinical risk factors linked to falls in older adults[4]. These guidelines concentrate on self-reported data on standard fall risk factors, captured as part of a clinical assessment. For each assessment the participant was asked the questions listed below, which were intended to capture the important risk factors covered by the guidelines:

1. Have you fallen in the last 12 months? *Y/N*
    a. If Y: How many times?
2. Have you had any problems walking or moving around? *Y/N*
3. Are you taking four or more prescription medications? *Y/N*
4. Do you have any problems with your feet? *Y/N*
5. Have you had any problems with your blood pressure dropping when you stand up? *Y/N*
6. Do you feel dizzy when you stand up from a sitting position? *Y/N*
7. Do you have any problems with your vision? *Y/N*
8. Have you had any change in your ability to manage your routine activities in the home? *Y/N*

Wearable sensor data

For each TUG test, participants were fitted with two wearable sensors (wireless inertial measurement units) which were attached by a user, using dedicated Velcro straps (or elasticated bandages if straps did not fit), to the mid-point of the left and right anterior shank (shin)[5]. Supplementary figure 1 details the setup. Participants were asked to complete the TUG test, '*as fast as safely possible*'. A standard chair with armrests was recommended. The timer was started the moment the clinician said 'go', and stopped the moment the participant's back touched the back rest of the chair. It was recommended that each participant be given time to become familiar with the test and that the test be demonstrated to them beforehand.

Wearable inertial sensors were oriented to capture movement about the anatomical medio-lateral axis. Inertial sensor data were simultaneously acquired from the two sensors via Bluetooth (QTUG™, Kinesis Health Technologies; Dublin, Ireland). Each sensor was tri-axial and contained an accelerometer and gyroscope. Sensor data were sampled at 102.4 Hz with a full scale range of 500 °/s and a sensitivity of 2 mV/°/s. Sensors were calibrated using a standard method[6]. The raw tri-axial gyroscope signals were low-pass filtered (zero-phase 2nd order Butterworth filter, 20 Hz corner frequency). Each test takes approximately five minutes, including application of the sensors and explaining the protocol.

Fall risk estimate

The AGS/BGS offer guidelines aimed to capture the main clinical risk factors linked to falls in older adults[4]. A logistic regression model was created using a number of the self-reported factors discussed in the AGS/BGS guidelines. History of falls in the past 5 years for each participant was obtained through a questionnaire and used as the target variable for each model. A fall was defined as *"an event which resulted in a person coming to rest on the lower level regardless of whether an injury was sustained, and not as a result of a major intrinsic event or overwhelming hazard"*[7]. Fall outcome data were verified using available hospital records as well as information provided by relatives.

The features included and used to classify participants according to falls history were as follows: gender, height, weight and age on date of assessment, polypharmacy, vision problems and orthostatic hypotension. A logistic regression model is used to produce a fall risk estimate ($FRE_{clinical}$); An estimate of the classifier performance of each model on unseen data was obtained using ten repetitions of ten-fold cross-validation[8].

The sensor-based fall risk estimate (FRE_{sensor}) method uses a subset of the QTUG mobility parameters applied to a regularized discriminant (RD) classifier model[9], with regularization parameter values set to λ=0.1 and r=0.1 prior to analysis. Ten repetitions of ten-fold cross validation[8] was used to estimate the generalized classifier performance. Using only the training data for each iteration of the cross-validation routine, a potential feature set was evaluated using a second inner cross-validation loop. Once a feature set is identified using the training data, it is tested using the withheld data for this iteration of the outer cross-validation loop[10], a process known as 'nested' cross-validation. Training and testing sets were randomly selected for each repetition.

The combined fall risk estimate ($FRE_{combined}$) is obtained by applying classifier combination theory[9] and averaging the posterior probabilities produced for a given participant by FRE_{sensor} and clinical $FRE_{clinical}$ to produce $FRE_{combined}$:

$$FRE_{combined} = \left(FRE_{sensor} + FRE_{clinical}\right)/2$$

The classifier performance for each model was estimated using Leave One Out (LOO) cross-validation, where N-1 samples were used to train the classifier model and the remaining sample used to test the performance, with this process repeated for each sample. The FRE_{sensor} feature and model selection was conducted using nested cross-validation as discussed above, where model selection is conducted using 10 repetitions of 10-fold cross-validation using only the training data, within the LOO procedure.

Frailty estimate

A logistic regression model with interaction terms included was used to calculate FE_{sensor}, using QTUG sensor features, combined with gender, age, height and weight, with a separate classifier model per gender. The features included in each model were selected using a cross-validated sequential forward feature selection procedure. Models were then evaluated using a separate repeated cross-fold validation, with 10 folds and 10 repetitions. Frailty was considered as a binary classification problem; grouping participants identified as frail and pre-frail (by the Fried phenotype criteria) together into one frail class and comparing this to a non-frail (robust under the Fried phenotype criteria) class. The output of this model was an estimate of the frailty category (frail/non-frail). This estimated frailty category was then compared to the true frailty category (as defined using modified Fried criteria[11]) to yield an estimate of the accuracy in classifying each participant according to frailty category. $FE_{clinical}$ and $FE_{combined}$ were calculated using the methodology outlined above for $FRE_{clinical}$ and $FRE_{combined}$.

Mobility impairment scores

The sensor data for each participant was processed using a previously reported algorithm[5,12] for assessment of gait and mobility. 59 features were calculated from the sensor data for each participant (known as the QTUG parameters), in order to characterize mobility.

Mobility issues are identified by grouping the 59 calculated mobility parameters into five functional categories: Speed, Variability, Symmetry, Transfers, Turning.

Mobility issues per functional category are identified by calculating a z-score calculated for each QTUG parameter per group; $z = \frac{x-\mu}{\sigma}$ where μ is population mean for a given parameter x, and σ is the population standard deviation. The population reference data are obtained from a previously reported independent sample[13]. The population data are stratified by gender to produce gender-specific values for population mean and standard deviation. The mean z-score per group is then calculated; if $|z\mu| \geq 2$, group is determined to be out of normal range. An estimate of the percentile

is calculated by applying the normal cumulative distribution function $P = \frac{1}{2}\left[1 = erf\left(\frac{x - \mu}{\sigma\sqrt{2}}\right)\right]$ to each parameter.

Positive parameters are defined as those for which a large value is considered to be a clinical indicator of good mobility (e.g. gait velocity), whereas a negative parameter is one where a large value is one where a large value is considered to be a clinical indicator of poor mobility (e.g. TUG time). A neutral parameter is then defined as one that does not fit into either category. If the mean mobility score (express as a percentage) for a category is above 70% the participant may have an impairment in that functional category.

Supplementary figures

Supplementary figure 1: Digital fall risk assessment protocol.

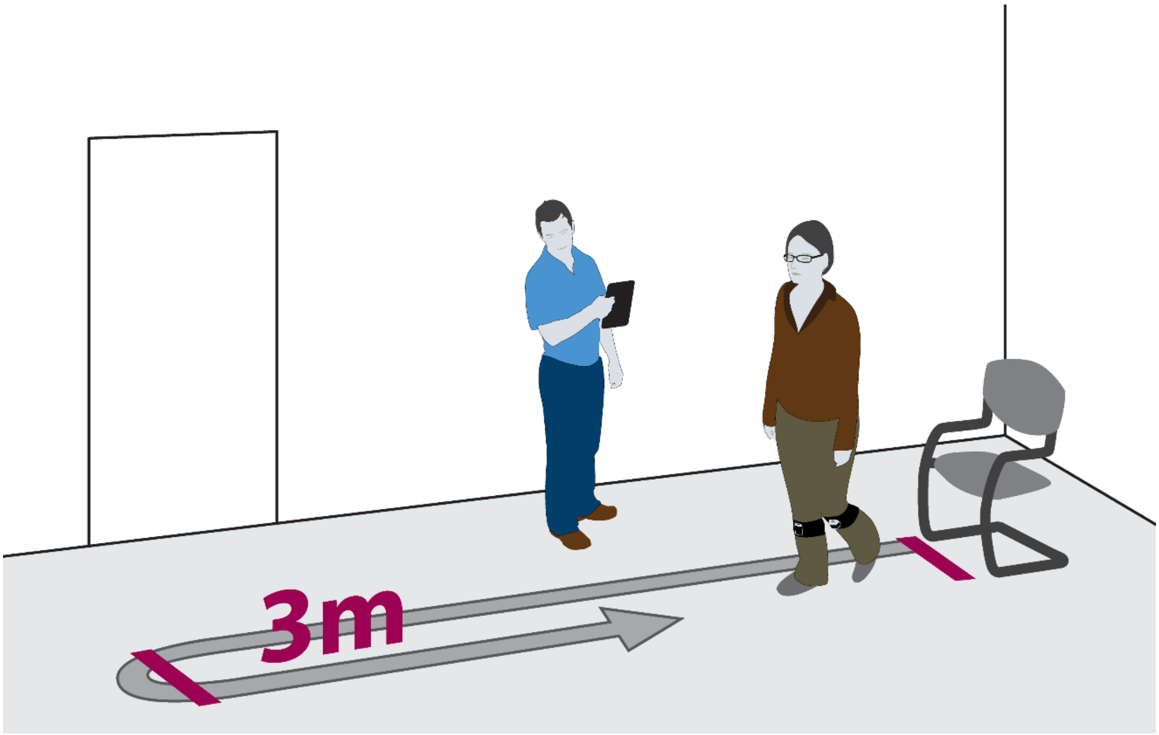

Supplementary figure 2: Prevalence of self-reported clinical risk factors per organization type

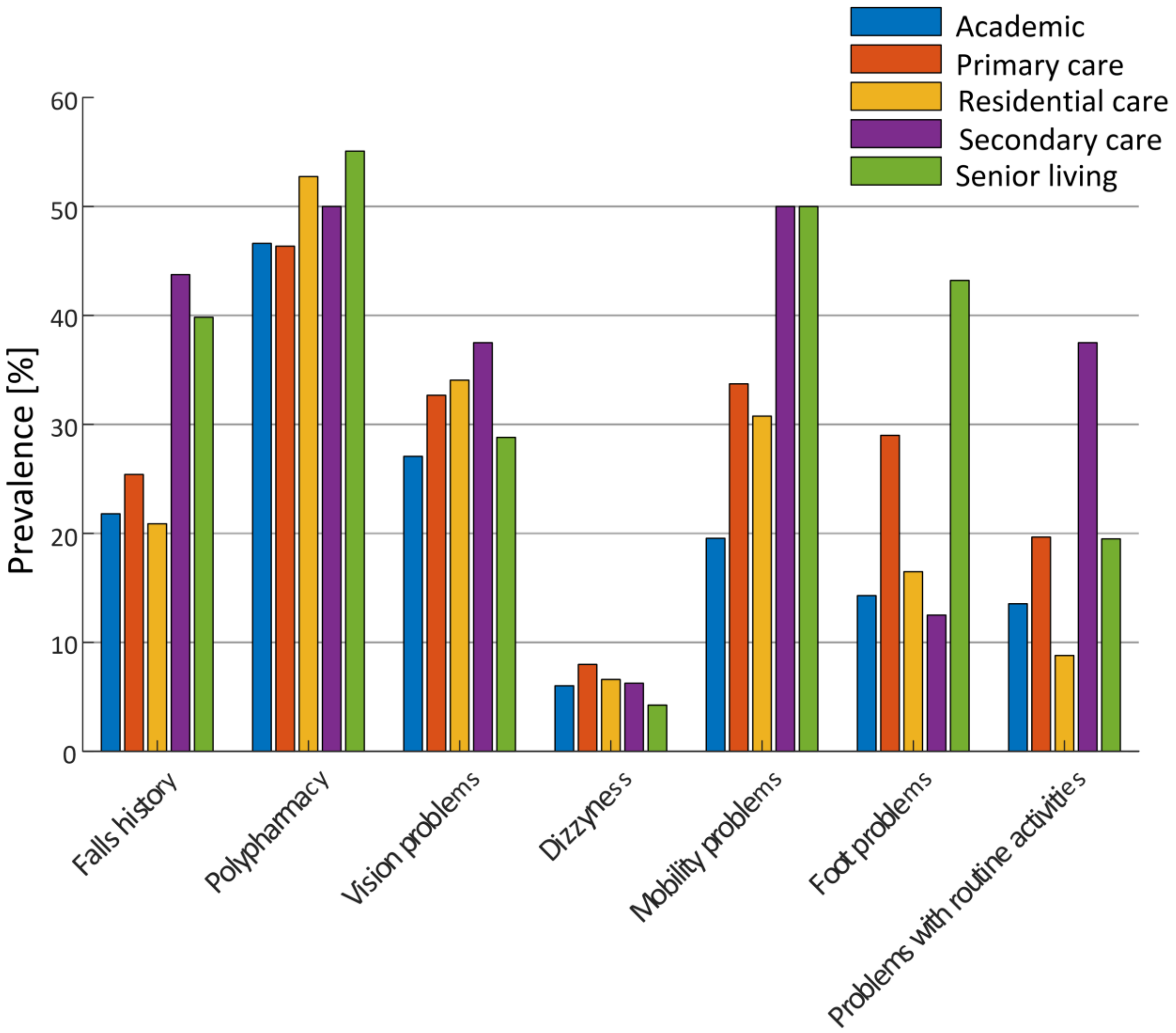

Supplementary figure 3: Prevalence of clinical risk factor by patient type

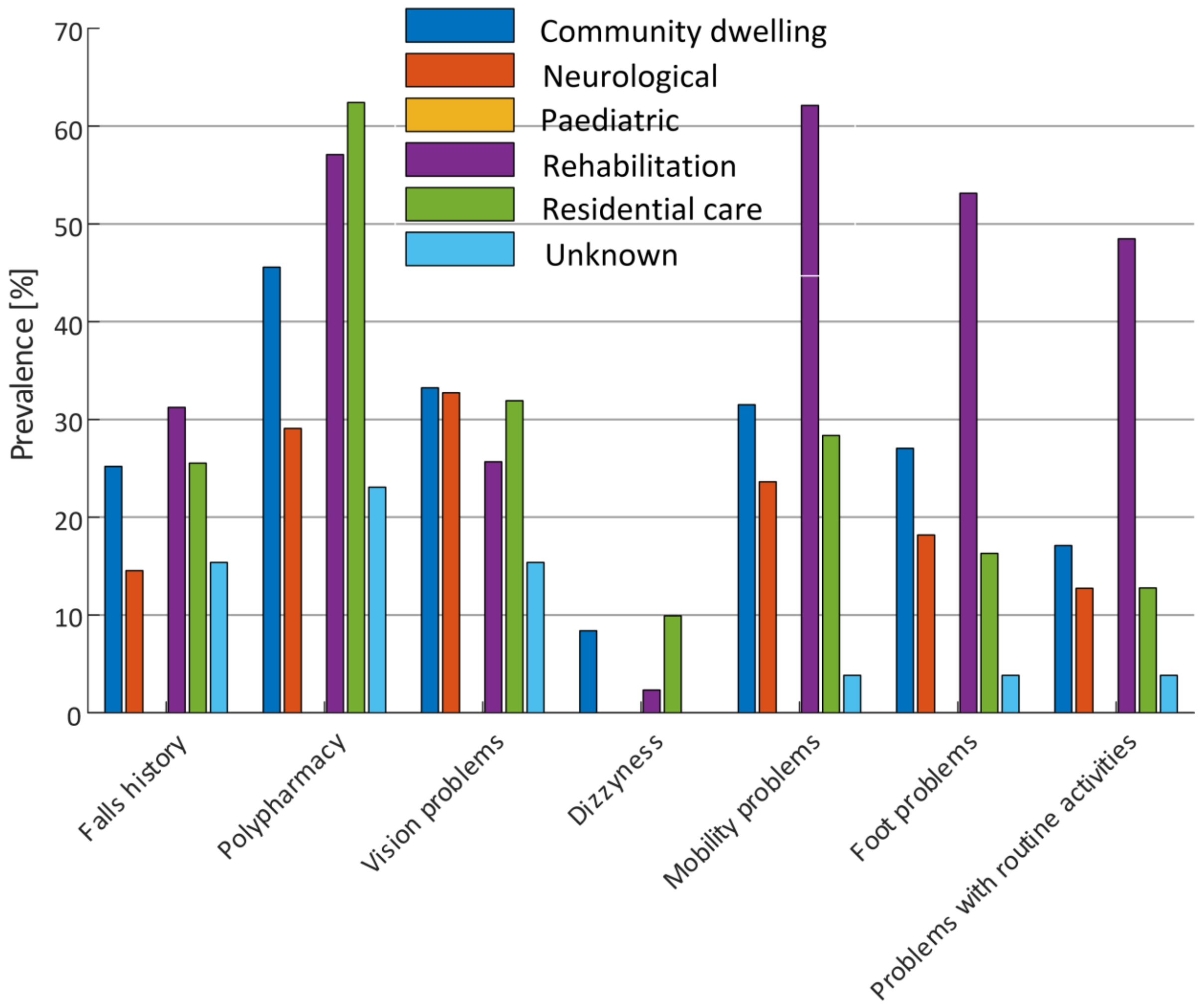

Supplementary figure 4: Mutual correlation (using Pearson's correlation coefficient) of falls risk, frailty and mobility impairment scores. $FRE_{combined}$ and $FE_{combined}$ are highly mutually correlated ($\rho \geq 0.7$), while Speed score, transfer score and turn score are moderately mutually correlated ($\rho \geq 0.5$)

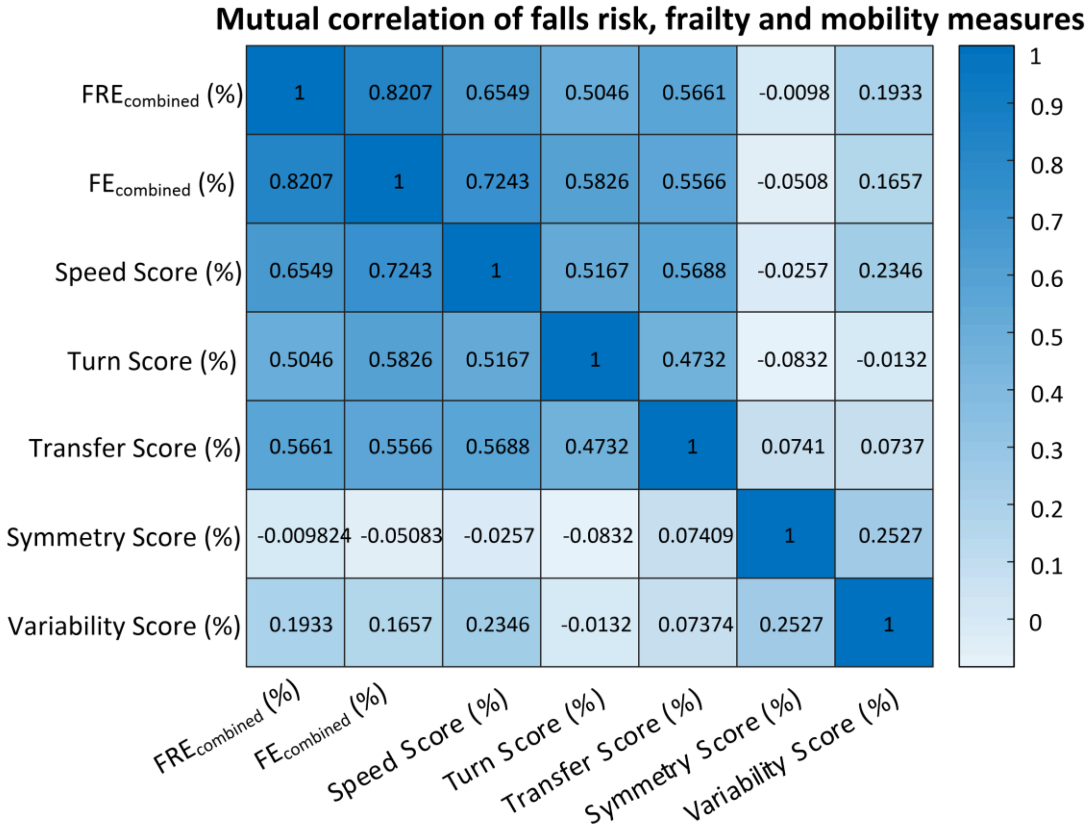

Supplementary figure 5: Association of frailty score ($FE_{combined}$) with falls history ($F=51.08$, $p<0.0001$). For each box, the central mark indicates the median, and the bottom and top edges of the box indicate the 25th and 75th percentiles, respectively. The whiskers extend to the most extreme data points not considered outliers, while outliers are denoted individually by '+'.

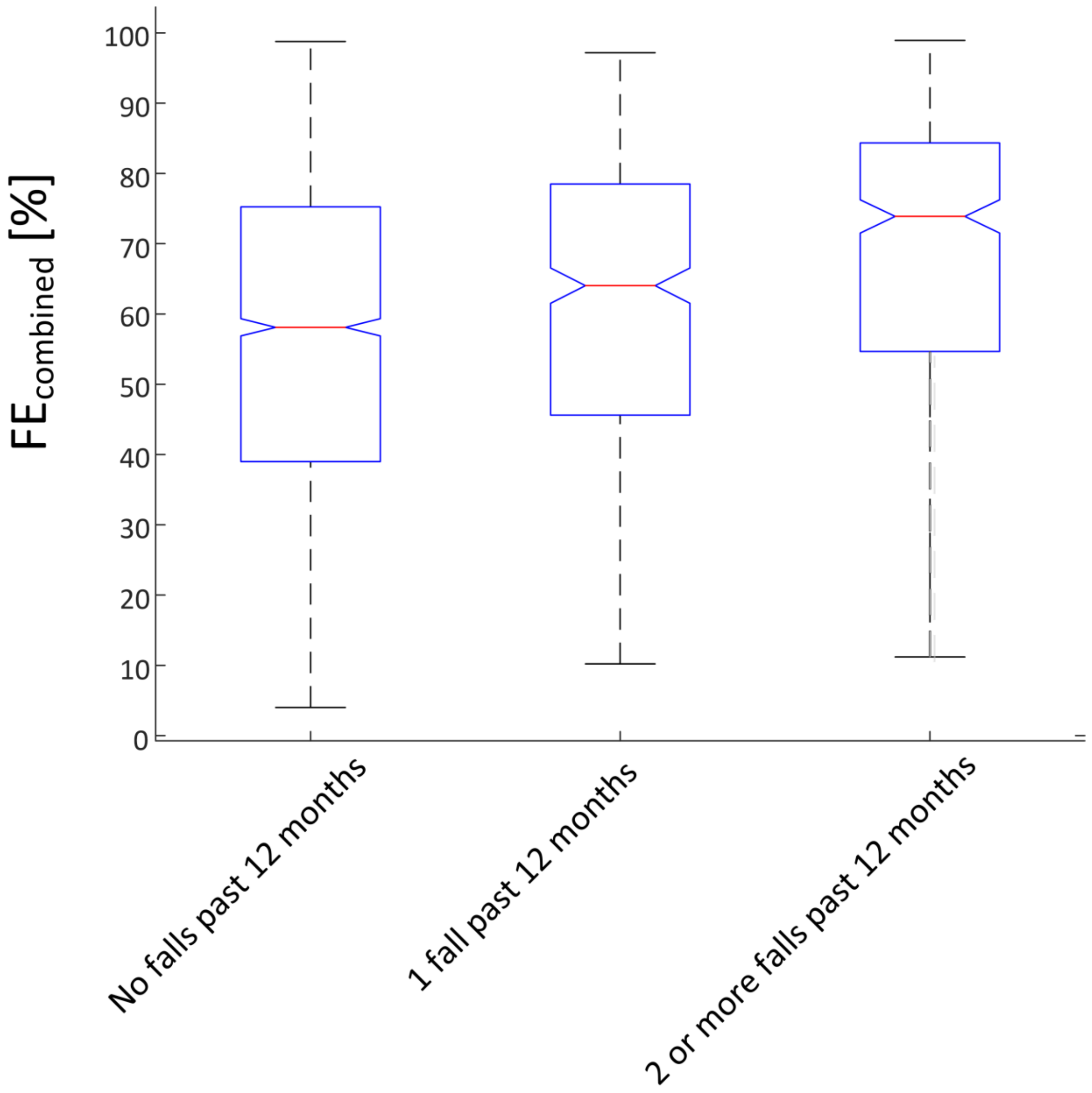

Supplementary figure 6: Association of mobility impairment scores with falls history. Each mobility score was significantly associated with falls history (speed: F=76.78, p<0.0001, turn: F=41.47, p<0.0001, transfers: F=115.46, p<0.0001, symmetry: F=4.13, p<0.05, variability: F=6.23, p<0.01.

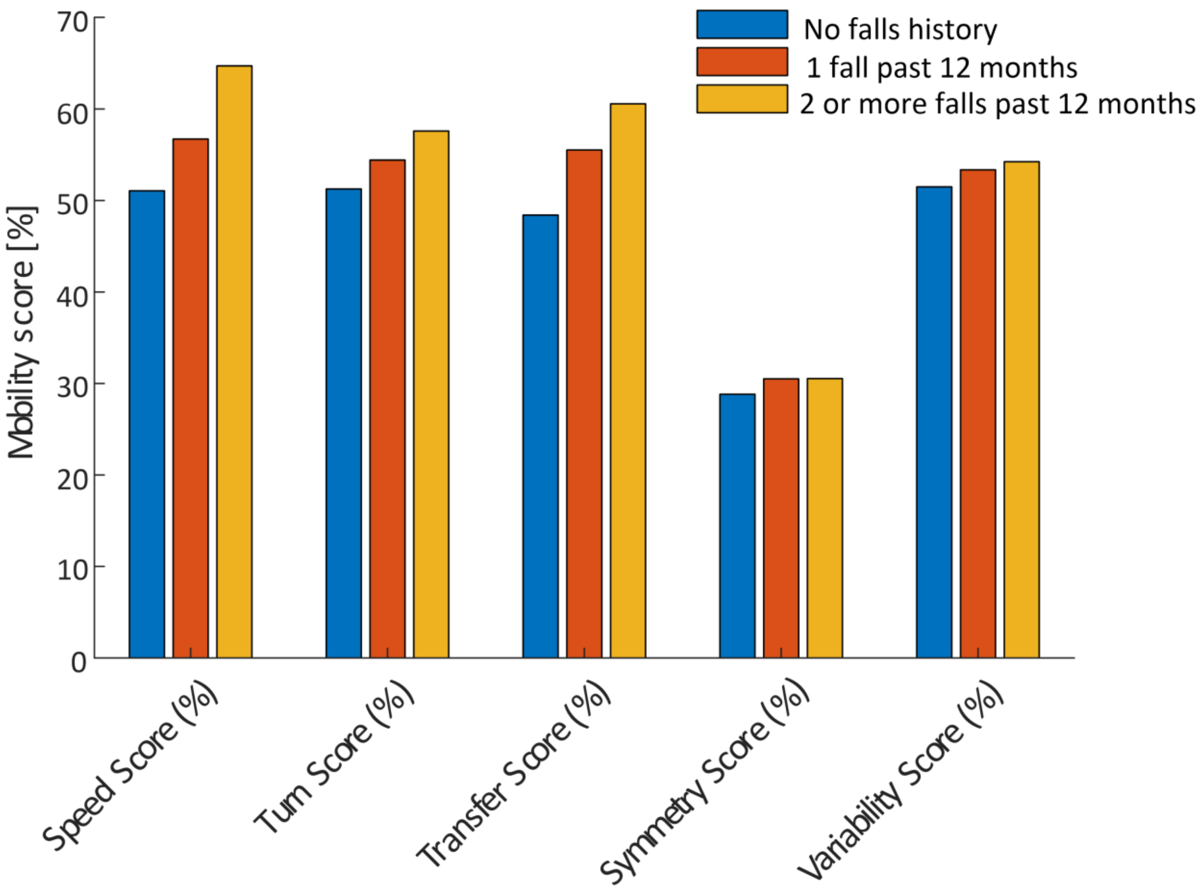

Supplementary figure 7: Association of mobility impairment scores with self-reported mobility problems. Four of five mobility scores were significantly associated with self-reported mobility problems (speed: $F=261.49$, $p<0.0001$, turn: $F=289.04$, $p<0.0001$, transfers: $F=547.07$, $p<0.0001$, symmetry: $F=3.24$, $p=0.07$, variability: $F=9.17$, $p<0.01$).

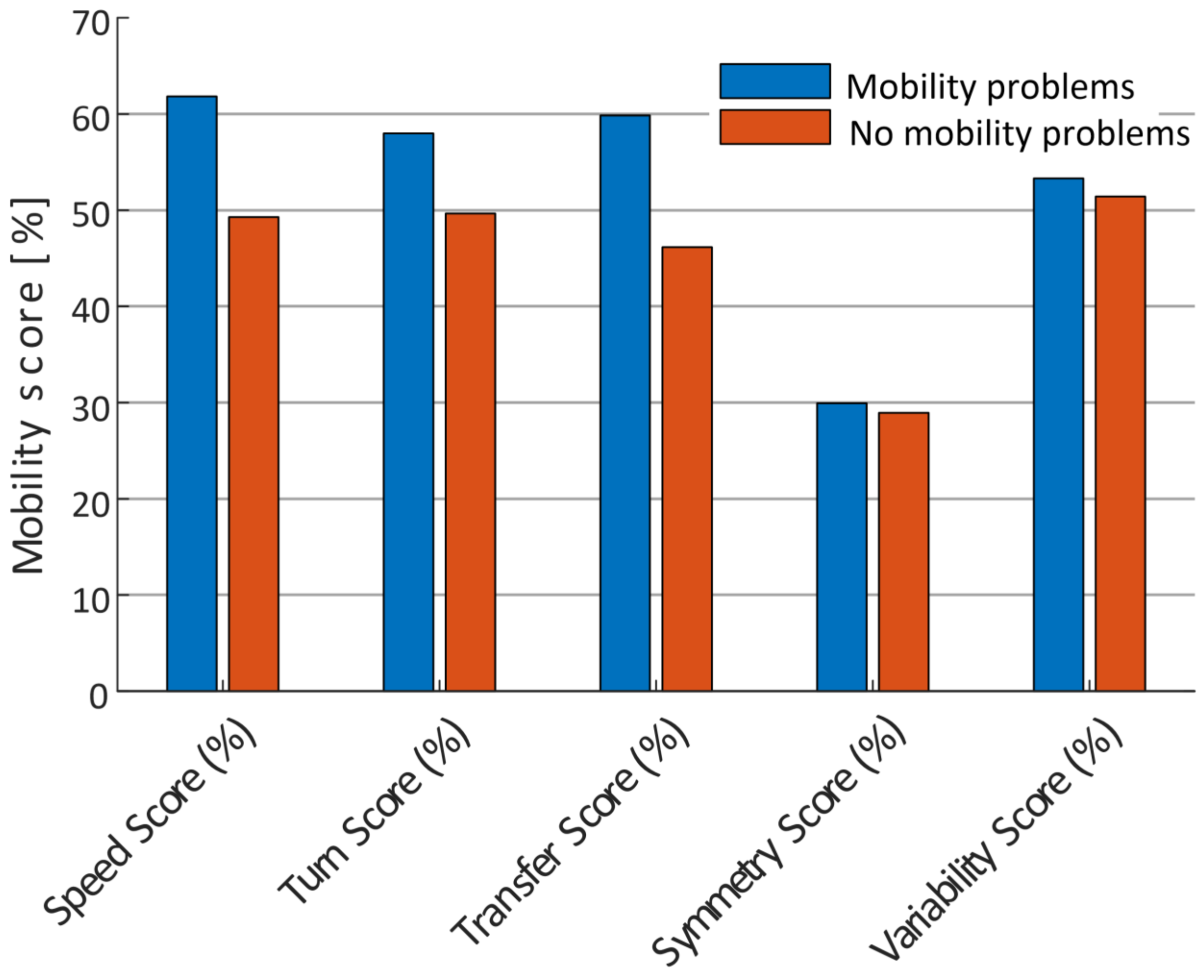

Supplementary references

1. Mathias S, Nayak U, Isaacs B. Balance in elderly patients: the "get-up and go" test. . *Arch Phys Med Rehabil.* 1986;67(6):387-389.
2. Podsiadlo D, Richardson S. The timed "Up & Go": a test of basic functional mobility for frail elderly persons. *J Am Geriatr Soc.* 1991;39:142-148.
3. Shumway-Cook A, Brauer S, Woollacott M. Predicting the probability for falls in community-dwelling older adults using the Timed Up & Go Test. *Phys Ther.* 2000;80:896 - 903.
4. Panel on Prevention of Falls in Older Persons AGS, British Geriatrics Society. Summary of the Updated American Geriatrics Society/British Geriatrics Society Clinical Practice Guideline for Prevention of Falls in Older Persons. *Journal of the American Geriatrics Society.* 2011;59(1):148-157.
5. Greene BR, O'Donovan A, Romero-Ortuno R, Cogan L, Ni Scanaill C, Kenny RA. Quantitative falls risk assessment using the timed up and go test. *IEEE Trans Biomed Eng.* 2010;57(12):2918-2926.
6. Ferraris F, Grimaldi U, Parvis M. Procedure for effortless in-field calibration of three-axis rate gyros and accelerometers. *Sens Mater.* 1995;7(5):311-330.
7. Tinetti ME, Speechley M, Ginter SF. Risk factors for falls among elderly persons living in the community. *N Engl J Med.* 1988;319(26):1701-1707.
8. Hastie T, Tibshirani R, Friedman JH. *The Elements of Statistical Learning.* 2nd ed: Springer; 2009.
9. Kuncheva LI. *Combining Pattern Classifiers; Methods and Algorithms.*: Wiley; 2004.
10. Kohavi R, John GH. Wrappers for feature subset selection. *Artificial Intelligence.* 1997;97(1-2):273-324.
11. Fried LP, Tangen CM, Walston J, et al. Frailty in Older Adults: Evidence for a Phenotype. *J Gerontol A Biol Sci Med Sci.* 2001;56(3):M146-157.
12. Greene BR, Redmond SJ, Caulfield B. Fall risk assessment through automatic combination of clinical fall risk factors and body-worn sensor data. *IEEE Journal of Biomedical and Health Informatics.* 2016;21(3):1-1.
13. Greene BR, Caulfield B. Objective assessment of functional mobility using the TUG test. IEEE Conference on Biomedical and Health Informatics (BHI) 2018 and the IEEE Conference on Body Sensor Networks (BSN) 2018 (BHI-BSN 2018); March 2018, 2018; Las Vegas NV.
